# Genetic Diversity of Polymorphic Marker Merozoite Surface Protein 1 (*Msp-1*) and 2 (*Msp-2*) Genes of *Plasmodium falciparum* Isolates From Malaria Endemic Region of Pakistan

**DOI:** 10.3389/fgene.2021.751552

**Published:** 2021-11-17

**Authors:** Shahid Niaz Khan, Rehman Ali, Sanaullah Khan, Muhammad Rooman, Sadia Norin, Shehzad Zareen, Ijaz Ali, Sultan Ayaz

**Affiliations:** ^1^ Department of Zoology, Faculty of Biological Sciences, Kohat University of Science and Technology, Kohat, Pakistan; ^2^ Department of Zoology, University of Peshawar, Peshawar, Pakistan; ^3^ Department of Zoology, Hazara University, Mansehra, Pakistan; ^4^ Department of Biosciences, COMSATS University Islamabad, Islamabad, Pakistan; ^5^ College of Veterinary Sciences and Animal Husbandry, Abdul Wali Khan University Garden Campus Mardan, Mardan, Pakistan

**Keywords:** *Plasmodium falciparum*, genetic diversity, polymorphism, msp-1 and msp-2 genes, nested PCR, Khyber Pakhtunkhwa, Pakistan

## Abstract

**Background:** Understanding the genetic diversity of *Plasmodium* species through polymorphic studies can assist in designing more effective control strategies of malaria like new drug formulation and development of a vaccine. Pakistan is moderate endemic for *Plasmodium falciparum*, but little is known about the genetic diversity of this parasite. This study aimed to investigate the molecular diversity of *P. falciparum* based on *msp-1* and *msp-2* genes in the malaria-endemic regions of Khyber Pakhtunkhwa, Pakistan.

**Methods:** A total of 199/723 blood samples, tested positive by microscopy for *falciparum* malaria, were collected from four districts (Dera Ismail Khan, Karak, Mardan, and Peshawar) of Khyber Pakhtunkhwa. Nested PCR amplification technique was employed to target block 2 of *msp-1* and the central domain of *msp-2* genes, including their respective allelic families K1, MAD20, RO33, FC27, and 3D7/IC, and to detect the extent of genetic diversity of *P. falciparum* clinical isolates.

**Results:** Among the 199 microscopy-positive *P. falciparum* samples, a total of 192 were confirmed using PCR. Ninety-seven amplicons were observed for *msp*-*1* and 95 for *msp-2*. A total of 33 genotypes, 17 for *msp-1* (eight K1, six MAD20, and three RO33) and 16 for *msp-2* (nine FC27 and seven 3D7/IC), were identified. The specific allelic frequency of the K1 family was higher (44.3%) than that of MAD20 (33.0%) and RO33 (23.0%) for *msp-1*, while the FC27 allelic family was dominant (60.0%) compared with 3D7/IC (40.0%) for *msp-2*. No polyclonal infection was observed in *msp-1* and *msp-2*. The expected heterozygosity was 0.98 and 0.97 for *msp-1* and *msp-2*, respectively.

**Conclusion:** It was concluded that the *P. falciparum* populations are highly polymorphic, and diverse allelic variants of *msp-1* and *msp-2* are present in Khyber Pakhtunkhwa, Pakistan.

## Introduction

Malaria causes 300–500 million cases worldwide and approximately 0.5–3 million deaths annually, the majority of which are caused by *Plasmodium falciparum* (Phillips, 2001; Ferreira et al., 2004). Pakistan is a moderate malaria-endemic region, and approximately 60% of its population is living in the endemic areas, whereas 177 million individuals are at risk of malaria (Qureshi et al., 2019). Annually, the estimated number of suspected and confirmed individuals is 3.5 million in Pakistan. The WHO included Pakistan as one of the six Eastern Mediterranean region countries with almost 100% population at risk of malaria (WHO, 2017; 2018). The endemicity of malaria varies in different provinces and even in different cities having variable climates. In 2017, about 30% of total malaria cases were reported from Khyber Pakhtunkhwa only, and the province has the highest reported cases of malaria (WHO, 2017).

The National Malaria Control Programmes (NMCP) has reported a record sixfold increase in *P. falciparum* during the last decade. The *falciparum* malaria has acknowledged sparse scientific attention, particularly concerning the molecular characterization of the local parasite population. The rise of *P. falciparum* in various districts of Pakistan may be attributable to the failed treatment of chloroquine resistance (Nizamani et al., 2006). Besides that, the heavy influx and continued presence of refugees from Afghanistan, where malaria is most prevalent, may contribute not only to the increasing number of cases of malaria but also to its genetic variations in Khyber Pakhtunkhwa province (Murtaza et al., 2004; Sheikh et al., 2005; Howard et al., 2011).

Analyses of the genotypes of *Plasmodium* spp. by PCR have remarkably improved our understanding of the biology of these parasites. In this regard, genetically distinct *P. falciparum* has been identified and extensively studied to further unveil its molecular epidemiology, parasite resistance, and potential vaccine candidates (Diggs et al., 1993; Greenhouse et al., 2006). The mainly used genetic markers of *P. falciparum* are the merozoite surface protein 1 (*msp-1*), merozoite surface protein 2 (*msp-2*), and glutamate-rich protein (*glurp*) (Smythe et al., 1991; Snounou and Beck, 1998). Allelic forms of these polymorphic markers have been reported in various parts of the world (Babiker et al., 1997; Jordan et al., 2001).

The *msp-1* and *msp-2* are antigenic proteins responsible for immunological responses in humans (Taylor et al., 1995; Aubouy et al., 2003). Block 2 of *msp-1* has three polymorphic allelic families identified as MAD20, K1, and RO33 (Contamin et al., 1996). Similarly, the central domain of the *msp-2* has two distinct families, i.e., 3D/IC and FC27 (Sallenave-Sales et al., 2000). These markers are unlinked and located on different chromosomes (Färnert et al., 2001). These features make them attractive candidates for studies where identification and enumeration of genetically distinct *P. falciparum* parasite subpopulations are of interest. As such, they have proven to be useful tools in molecular epidemiology studies in different epidemiological settings as well as to distinguish treatment failures from new infections in antimalarial drug trials (Cattamanchi et al., 2003; Collins et al., 2006).

The genetic diversity of *P. falciparum* population is an important indicator of the malaria transmission intensity in an area (Babiker et al., 1995; Paul et al., 1998). A high endemic area is generally characterized by extensive parasite diversity, and infected humans often carry multiple genotypes. Conversely, the parasite population in a low transmission area has a limited genetic diversity, and most infections are monoclonal (Babiker et al., 1997; Haddad et al., 1999; Peyerl-Hoffmann et al., 2001; Gómez et al., 2002). The *P. falciparum* field isolates have been characterized in Afghanistan, Iran, and India and previously from Sindh and Baluchistan in Pakistan, using the above-mentioned molecular markers. Therefore, we investigated the genetic diversity and polymorphic nature of *P. falciparum* isolates in selective districts of the malaria endemic province Khyber Pakhtunkhwa of Pakistan.

## Materials and Methods

### Ethics and Consent for Participation

The study protocol was approved by the Institutional Ethical Review Committee of Kohat University of Science and Technology (KUST), Kohat-26000, Pakistan. Signed and written informed consent was obtained from the participants/legal guardians before sample collection.

### Sample Collection and Analysis

Blood samples were randomly collected from suspected individuals ≥1 year at the Malaria Control Laboratories of District Headquarters Hospitals (DHQs) in four districts of Khyber Pakhtunkhwa (Dera Ismail Khan, Karak, Peshawar, and Mardan). A total of 723 suspected individuals with fever or history of fever were screened. Finger-pricked blood was collected on a glass-slide to prepare thick and thin blood smears, air-dried, and stained with Giemsa’s stain (10%) for 15 min. The slides were examined under a microscope (Olympus CX31, Tokyo, Japan) by experienced laboratory technicians for *Plasmodium* species-specific identification. After careful examination, blood smears were considered negative when no parasite was detected and vice versa. Among the screened individuals, a total of 199 were confirmed positive for *P. falciparum* infection by microscopy.

Additionally, 200 μl of blood was collected into EDTA tubes, labelled, and transferred to the Molecular Parasitology and Virology Laboratory, Department of Zoology, KUST, Kohat-26000, Khyber Pakhtunkhwa, Pakistan, and stored at −80°C in a low deep freezer until genomic DNA extraction. Furthermore, a brief epidemiological/demographic history was also recorded using a structured questionnaire. The demographic data will be published elsewhere.

### DNA Extraction and PCR Amplification

Genomic DNA was extracted from blood using the GF1 DNA extraction kit (Vivantis, Shah Alam, Selangor). Nested PCR was recruited to determine *Plasmodium* species with the help of 18S rRNA amplification using primers rPLUf: 5′-TTA​AAA​TTG​TTG​CAG​TTA​AAA​CG-3′ and rPLUr: 5′-CCT​GTT​GTT​GCC​TTA​AAC​TTC-3′ as previously described (Snounou et al., 1993; Singh et al., 1999). The amplicons were used as a template in the nested PCR; and species-specific primers rFALf: 5′-TTA​AAC​TGG​TTT​GGG​AAA​ACC​AAA​TAT​ATT-3′ and rFALr: 5′-ACA​CAA​TGA​ACT​CAA​TCA​TGA​CTA​CCC​GTC-3′ were employed for *P. falciparum* detection (Snounou et al., 1993). Deionized water and genomic DNA from laboratory strains were used as negative and positive control, respectively. Both primary and secondary PCRs were carried out in a final volume of 20 μl containing 5.0 μl of DNA, 0.4 μl of 10 mM of each dNTP (Fermentas, Hanover, MD, USA), 2.4 μl of 10× PCR buffer, 1.5 μl of 25 mM MgCl_2_, 0.5 μl of 5 U/μl *Taq* DNA polymerase (Fermentas, Hanover, MD, USA), paired primers of 1.0 μl each (20 μM), and 8.2 μl of sterile water. Thermocycler (Nyxtech, Boston, MA, USA) was used to complete all the reactions under the following conditions for primary (35 cycles) and secondary PCR (30 cycles): initial denaturation at 94°C for 1 min, extension at 94°C for 1 min, 58°C for 2 min and 72°C for 5 min, and final elongation at 72°C for 5 min.

The *msp-1* and *msp-2* genes were amplified using specific primers as per standard protocol previously described (Snounou et al., 1999). The primary reaction used a set of primers corresponding to the conserved regions of block 2 for *msp-1* and block 3 for *msp-2*. The second reaction primer set targets specific allelic families of *msp-1* (KI, MAD20, and RO33) or *msp-2* (3D7/IC and FC27). The cycling conditions for both *msp-1* and *msp-2* as well as primers were previously described (Somé et al., 2018).

Agarose gel (2%) stained with ethidium bromide was used to evaluate the PCR products under UV illumination. A 50 and 100-bp DNA ladders (Promega, Madison, WI, USA) were used; and alleles of *msp-1* and *msp-2* were categorized according to their molecular weights.

### Statistical Analysis

Primarily, Microsoft Excel was used to manage the data. Statistical analyses were performed using SPSS (Version 20). The allelic frequencies for *msp-1* and *msp-2* were calculated and expressed in percentages. The proportion of alleles observed at each locus was compared using a chi-square test. The expected heterozygosity (H_e_) was calculated using the following formula: H_e_ = [*n*/(*n* − 1)] [(1 − Σ*p*_*i*_^2^)], where *n* is the number of isolates sampled and *p*_*i*_ is the allele frequency at a given locus (Nei, 1978). The *p*-value (0.05) was assumed to be statistically significant.

## Results

**FIGURE 1:**
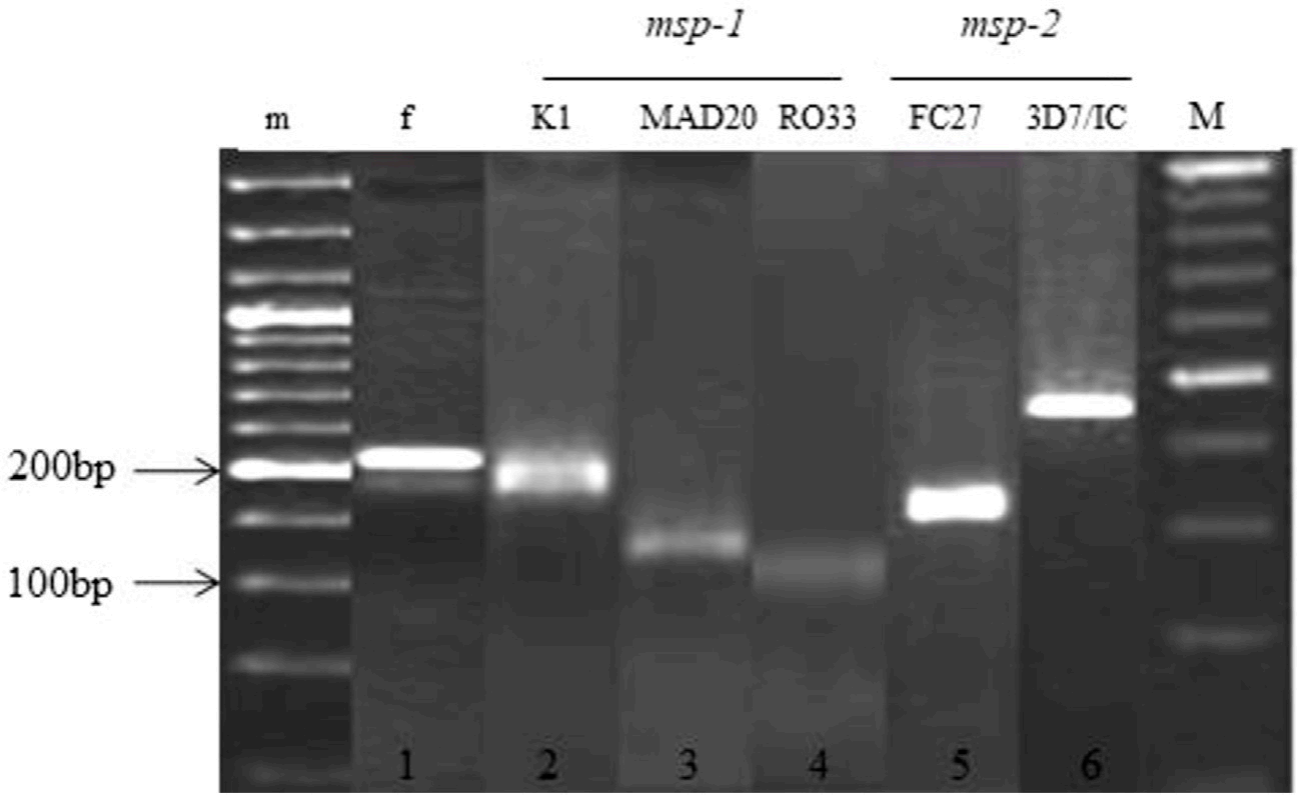
Allelic families of *msp-1* and *msp-2* genes of *Plasmodium falciparum* 1 show the positive infection of *Plasmodium falciparum*; 2, 3, and 4 represent the corresponding alleles of *msp-1*; 5 and 6 show the alleles of *msp-2*; m is a 50-bp DNA ladder marker, and M is a 100-bp DNA ladder marker.

Out of 199 microscopy-positive samples, 192 were further confirmed for *P. falciparum* using PCR, whereas seven samples (3.2%) were excluded due to negative PCR outcome (Figure 1).

Out of total, 97 amplicons were observed for *msp*-*1* and 95 for *msp-2*. In *msp-1*, the highest frequency of K1, MAD20, and RO33 was 44.3% (43/97), 33.0% (32/97), and 23.0% (22/97), respectively (Table 1). In *msp-2*, the FC27 has the highest frequency of 60.0% (57/95), followed by 3D7/IC with 40.0% (38/95) (Table 2). However, no polyclonal infection was observed in *msp-1* and *msp-2*.

**TABLE 1:** Genotyping of *Plasmodium falciparum msp-1* in Khyber Pakhtunkhwa.

MSP-1 (n = 97)	Size (bp)	Frequency (%)	No. of alleles	*p*-Value^a^
K1	180–250	43 (44.3)	8	0.994
MAD20	110–250	32 (33.0)	6	
RO33	130–190	22 (23.0)	3	

Note. bp, base pair.

a The *p*-value represents significance level of allelic variants among different districts.

**TABLE 2:** Genotyping of *Plasmodium falciparum msp-2* in Khyber Pakhtunkhwa.

MSP-2 (n = 95)	Size (bp)	Frequency (%)	No. of alleles	*p-*Value^a^
3D7/IC	400–580	38 (40.0)	9	0.963
FC27	300–430	57 (60.0)	7	

Note. bp, base pair.

a The *p*-value represents significance level of allelic variants among different districts.

Seventeen alleles were detected in *msp-*1 comprising eight alleles (180–350 bp) for K1, six alleles (110–250 bp) for MAD20, and three alleles (130–190 bp) for RO33. However, 16 alleles were attributed to *msp-2*, with nine alleles (400–580 bp) for FC27 and seven alleles (300–430 bp) for 3D7/IC. The district-wise details of allelic frequencies are presented in the supplementary materials (Supplementary Tables S1, S2). The expected heterozygosity was 0.98 and 0.97 for *msp-1* and *msp-2*, respectively.

## Discussion

Molecular studies provide an insight into the transmission intensity and genetic variation of parasite population within a region. The genetic diversity may be linked with the cross-border movement of populations living in the Frontier Regions of Pakistan (Ghanchi et al., 2010; Zakeri et al., 2010). Usually, areas with high malaria transmission are observed to have an extensive genetic diversity (Paul et al., 1998; Peyerl-Hoffmann et al., 2001). This study provides the basis to explore the genetic diversity of *P. falciparum* in the endemic regions of Khyber Pakhtunkhwa.

In the present study, the number of successfully genotyped samples for *msp-1* was higher as compared with that for *msp-2.* This result is consistent with studies reported from Cote d’Ivoire and Gabon (Yavo et al., 2016) and Burkina Faso (Soulama et al., 2009; Somé et al., 2018). However, in contrast, Mohammad et al. (2019) from Ethiopia, Soe et al. (2017) from Myanmar, and A-Elbasit et al. (2007) from Sudan reported a relatively high frequency of *msp-2* genotyped samples. Furthermore, of 192 positive samples for *P. falciparum*, less than half showed an amplicon for *msp-1* (n = 97) or *msp-2* (n = 95). In previous studies, relatively higher numbers of amplicons were observed for *msp-1* or *msp-2* (Somé et al., 2018; Mohammed et al., 2019; Eltayeb et al., 2020; Papa Mze et al., 2020). The nonspecific binding during *P. falciparum* identification by PCR may justify the smaller number of amplicons for *msp-1* and *msp-2* in the current study.

It was observed that 17 allelic variants of *msp-1* and 16 of *msp-2* were present in the studied areas. This result is in comparison with a similar study from the south of Pakistan (Ghanchi et al., 2010), Iran, South Africa, Myanmar, Sudan, Senegal, and Thailand (Heidari et al., 2007; Soe et al., 2017; Somé et al., 2018; Ndiaye et al., 2019; Eltayeb et al., 2020). However, in two southern districts Bannu and Kohat of Khyber Pakhtunkhwa, less allelic variants of *P. falciparum* were reported (Khatoon et al., 2010; Khatoon et al., 2012). Similarly, a study from the hypo-endemic area of Colombia reported only one allele of *msp-1* and three alleles of *msp-2* (Montoya et al., 2003). This difference might be due to low malaria endemicity since higher allele frequencies have been reported with high malaria transmission (Konaté et al., 1999; Soulama et al., 2009), suggesting that malaria endemicity affects the circulating strain number. However, high genetic diversity was observed in the Kingdom of Eswatini, which is regarded as a low transmission area for *P. falciparum* (Roh et al., 2019). Therefore, the genetic diversity may also be attributed to the effects of several factors such as indiscriminate use of long-lasting insecticide-treated nets (LLINs), indoor insecticide spraying, and antimalarial pressure (Soulama et al., 2009).

The K1 and FC27 alleles of *msp-1* and *msp-2* were predominant, respectively. The K1 and FC27 allelic families were previously reported from Kohat district (Khatoon et al., 2012). Nevertheless, the present findings also showed slight discrepancy with the previous studies (Zakeri et al., 2005; Ghanchi et al., 2010). The current study and previous findings suggest that K1 and FC27 allelic families might have prominent roles in clinical malaria at least in southern Khyber Pakhtunkhwa. However, further in-depth investigation with larger dataset should be carried out to unveil the genetic diversity and prevailing genotypes.

Interestingly, no polyclonal infection was detected in the present study. Malaria treatment policies, geographic isolation, and transmission intensities may result in spatial heterogeneity of *P. falciparum* (Khatoon et al., 2010). Most importantly, the use of highly potent antiplasmodial drugs that kill the asexual blood stage parasites and gametocytes are more likely to decrease parasite transmission and clonal diversity (Targett et al., 2001; Greenwood et al., 2008). However, in the adjacent districts (Bannu and Kohat) of Khyber Pakhtunkhwa, polyclonal infections were reported previously (Khatoon et al., 2010; Khatoon et al., 2012). It is worth mentioning that these two districts (Bannu and Kohat) accommodated one million internally displaced persons (IDPs) from tribal areas of North Waziristan Agency (NWA) sharing its border with Afghanistan, which may have introduced new genetically distinct variants of *P. falciparum*. It shows that the huge influx and migration of people between the study area and the neighboring countries like Iran and especially Afghanistan may introduce different alleles of *P. falciparum* into Khyber Pakhtunkhwa province of Pakistan.

Malaria transmission in Pakistan is markedly seasonal and prone to outbreaks, in particular geographical areas, especially Khyber Pakhtunkhwa, Baluchistan, and Sindh province (Yasinzai and Kakarsulemankhel, 2009). Pakistan is considered to be endemic for malaria, but the precise data on the genetic diversity of malaria in Pakistan are still lacking (Khan et al., 2005). As limitations, a small number of samples were amplified for *msp-1* and *msp-2*, and the use of nested PCR instead of DNA sequencing could possibly underestimate the genetic diversity. Therefore, studies with larger datasets and more robust techniques should be used to explore the genetic diversity in the future. Furthermore, only microscopy-positive samples for *P. falciparum* were further subjected to nested PCR, which is another limitation of the present study.

## Conclusion

It was concluded that extensively diverse and polymorphic *P. falciparum* populations of merozoite surface protein 1 (*msp-1*) and 2 (*msp-2*) are present in Khyber Pakhtunkhwa, Pakistan.

## Data Availability

The datasets presented in this study can be found in online repositories. The names of the repository/repositories and accession number(s) can be found in the article/Supplementary Material.
