# Supplementary material for: Genetic Diversity of Polymorphic Marker Merozoite Surface Protein 1 (Msp-1) and 2 (Msp-2) Genes of Plasmodium falciparum Isolates From Malaria Endemic Region of Pakistan
Source: Front Genet. 2021 Nov 17;12:751552. doi: 10.3389/fgene.2021.751552 (PMC8635745; doi:10.3389/fgene.2021.751552)
Supplement: Supplementary file 1 [file Table1.DOCX]

**Supplementary Table 1: Distribution of different allele types of *P. falciparum* *msp-1* in the selective districts of Khyber Pakhtunkhwa**

| **MSP-1** | **Size (bp)** | **D. I. Khan n (%)** | **Karak n (%)** | **Mardan n (%)** | **Peshawar n (%)** | **Total n (%)** | ***p-*value** |
| --- | --- | --- | --- | --- | --- | --- | --- |
| K1 | 180-250 | 09 (9.3) | 11 (11.3) | 10 (10.3) | 13 (13.4) | 43 (44.3) | 0.994 |
| MAD20 | 110-250 | 08 (8.2) | 07 (7.2) | 07 (7.2) | 10 (10.3) | 32 (33.0) |  |
| RO33 | 130-190 | 05 (5.1) | 04 (4.1) | 06 (6.2) | 07 (7.2) | 22 (23.0) |  |
| Total |  | 22 (23.0) | 22 (23.0) | 23 (24.0) | 30 (31.0) | 97 (100) |  |

Key: *n*= number of alleles
