# Supplementary material for: Genetic Diversity of Polymorphic Marker Merozoite Surface Protein 1 (Msp-1) and 2 (Msp-2) Genes of Plasmodium falciparum Isolates From Malaria Endemic Region of Pakistan
Source: Front Genet. 2021 Nov 17;12:751552. doi: 10.3389/fgene.2021.751552 (PMC8635745; doi:10.3389/fgene.2021.751552)
Supplement: Supplementary file 2 [file Table2.DOCX]

**Supplementary Table 2: Distribution of different allele types of *P. falciparum msp*-2 in the selective districts of Khyber Pakhtunkhwa**

| **MSP-2** | **Size (bp)** | **D. I. Khan n (%)** | **Karak n (%)** | **Mardan n (%)** | **Peshawar n (%)** | **Total n (%)** | ***p-*value** |
| --- | --- | --- | --- | --- | --- | --- | --- |
| 3D7/IC | 400-580 | 09 (9.5) | 09 (9.5) | 11 (12.0) | 09 (9.5) | 38 (40.00) | 0.963 |
| FC27 | 300-430 | 11 (12.0) | 14 (15.0) | 17 (18.0) | 15 (16.0) | 57 (60.00) |  |
| Total |  | 20 (21.0) | 23 (24.2) | 28 (29.5) | 24 (25.2) | 95 (100) |  |

Key: *n*= number of alleles
